# Homeostasis of Second Messenger Cyclic-di-AMP Is Critical for Cyanobacterial Fitness and Acclimation to Abiotic Stress

**DOI:** 10.3389/fmicb.2018.01121

**Published:** 2018-05-29

**Authors:** Marco Agostoni, Alshaé R. Logan-Jackson, Emily R. Heinz, Geoffrey B. Severin, Eric L. Bruger, Christopher M. Waters, Beronda L. Montgomery

**Affiliations:** ^1^Cell and Molecular Biology Graduate Program, Michigan State University, East Lansing, MI, United States; ^2^Department of Energy Plant Research Laboratory, Michigan State University, East Lansing, MI, United States; ^3^Department of Microbiology and Molecular Genetics, Michigan State University, East Lansing, MI, United States; ^4^Department of Biochemistry and Molecular Biology, Michigan State University, East Lansing, MI, United States

**Keywords:** abiotic stresses, c-di-AMP, c-di-GMP, cyanobacteria, ionic stress, osmotic stress, salt stress, second messengers

## Abstract

Second messengers are intracellular molecules regulated by external stimuli known as first messengers that are used for rapid organismal responses to dynamic environmental changes. Cyclic di-AMP (c-di-AMP) is a relatively newly discovered second messenger implicated in cell wall homeostasis in many pathogenic bacteria. C-di-AMP is synthesized from ATP by diadenylyl cyclases (DAC) and degraded by specific c-di-AMP phosphodiesterases (PDE). C-di-AMP DACs and PDEs are present in all sequenced cyanobacteria, suggesting roles for c-di-AMP in the physiology and/or development of these organisms. Despite conservation of these genes across numerous cyanobacteria, the functional roles of c-di-AMP in cyanobacteria have not been well-investigated. In a unique feature of cyanobacteria, phylogenetic analysis indicated that the broadly conserved DAC, related to CdaA/DacA, is always co-associated in an operon with genes critical for controlling cell wall synthesis. To investigate phenotypes regulated by c-di-AMP in cyanobacteria, we overexpressed native DAC (*sll0505*) and c-di-AMP PDE (*slr0104*) genes in the cyanobacterium *Synechocystis* sp. PCC 6803 (hereafter *Synechocystis*) to increase and decrease intracellular c-di-AMP levels, respectively. DAC- and PDE-overexpression strains, showed abnormal aggregation phenotypes, suggesting functional roles for regulating c-di-AMP homeostasis *in vivo*. As c-di-AMP may be implicated in osmotic responses in cyanobacteria, we tested whether sorbitol and NaCl stresses impacted expression of *sll0505* and *slr0104* or intracellular c-di-AMP levels in *Synechocystis*. Additionally, to determine the range of cyanobacteria in which c-di-AMP may function, we assessed c-di-AMP levels in two unicellular cyanobacteria, i.e., *Synechocystis* and *Synechococcus elongatus* PCC 7942, and two filamentous cyanobacteria, i.e., *Fremyella diplosiphon* and *Anabaena* sp. PCC 7120. C-di-AMP levels responded differently to abiotic stress signals in distinct cyanobacteria strains, whereas salt stress uniformly impacted another second messenger cyclic di-GMP in cyanobacteria. Together, these results suggest regulation of c-di-AMP homeostasis in cyanobacteria and implicate a role for the second messenger in maintaining cellular fitness in response to abiotic stress.

## Introduction

Cyanobacteria comprise a group of highly diverse, oxygenic photosynthetic bacteria that respond to a range of abiotic and biotic signals in their environment, from light that has direct impacts on photosynthesis and productivity to osmotic and saline stresses. These organisms are highly abundant in many ecosystems (Garcia-Pichel et al., 2003) and as carbon, and sometimes nitrogen fixers, contribute significantly to global carbon and nitrogen cycles. Second messengers are critical intracellular molecules that are regulated in response to specific external stimuli known as first messengers. Control of second messenger homeostasis is used frequently to initiate physiological changes that occur in microorganisms as a part of environmental acclimation. A range of second messengers have been identified that play key roles in regulating environmentally-controlled physiological responses in cyanobacteria (Agostoni and Montgomery, 2014). Among second messengers, cyanobacteria commonly rely on cyclic nucleotide signaling molecules such as cyclic AMP (i.e., cAMP) (Ohmori et al., 1988, 2001, 2002; Katayama and Ohmori, 1997; Terauchi and Ohmori, 1999, 2004; Ohmori and Okamoto, 2004; Okamoto et al., 2004; Imashimizu et al., 2005) and cyclic GMP (i.e., cGMP) (Ochoa De Alda et al., 2000; Cadoret et al., 2005). However, dicyclic nucleotides such as cyclic dimeric GMP (hereafter, cyclic di-GMP or c-di-GMP) have only recently been reported in these organisms. In cyanobacteria, c-di-GMP has roles in acclimation to light, phototaxis, and cellular aggregation (Savakis et al., 2012; Agostoni et al., 2013, 2016; Enomoto et al., 2014, 2015; Angerer et al., 2017).

The second messenger cyclic dimeric AMP (hereafter, cyclic di-AMP or c-di-AMP) is a relatively newly discovered cyclic dinucleotide (Romling, 2008; Witte et al., 2008; Fu et al., 2016; Jenal et al., 2017; Krasteva and Sondermann, 2017). Cyclic di-AMP is synthesized by diadenylyl cyclase (DAC; PF02457) from two molecules of ATP and degraded by specific phosphodiesterase (PDE) enzymes into pApA (Corrigan and Grundling, 2013). Cyclic di-AMP and its functional roles *in vivo* have been studied primarily in Gram-positive bacteria. In Gram-positive species, the regulation of c-di-AMP homeostasis has been associated with a range of responses. Cyclic di-AMP levels impact growth (Witte et al., 2013; Rismondo et al., 2016; Whiteley et al., 2017), sporulation (Oppenheimer-Shaanan et al., 2011; Mehne et al., 2014; Zheng et al., 2015; Raguse et al., 2017), virulence (Bai et al., 2013; Cho and Kang, 2013; Du et al., 2014; Yang et al., 2014; Dey et al., 2015), biofilm formation related to host–microbe interactions (Townsley et al., 2018), and DNA repair damage responses or the coordination of DNA damage response and stress homeostasis (Bejerano-Sagie et al., 2006; Witte et al., 2008; Gándara and Alonso, 2015; Gándara et al., 2017; Raguse et al., 2017), among other phenotypes in a range of Gram-positive strains. A role for c-di-AMP in growth and virulence has also been observed for the Gram-negative *Borrelia burgdorferi* and *Chlamydia trachomatis* (Barker et al., 2013; Ye et al., 2014). Additionally, c-di-AMP has been implicated in cellular responses to abiotic stresses in multiple Gram-positive bacteria (Dengler et al., 2013; Bowman et al., 2016; Zhu et al., 2016).

Whereas numerous roles for c-di-AMP have been documented in Gram positive bacteria, limited insights into the roles of this molecule in other bacteria have been reported. Notably, cyanobacteria have recently been reported to contain c-di-AMP synthesis genes (Agostoni and Montgomery, 2014) and c-di-AMP accumulation has recently been reported in *Synechococcus elongatus* sp. PCC 7942 (Rubin et al., 2018). All sequenced cyanobacteria possess at least one DAC, with some exceptions of strains that carry two DACs (Agostoni and Montgomery, 2014). Unlike those DAC proteins reported in many bacteria which include a DAC domain with fusions to other regulatory domains, the DACs of cyanobacteria generally contain only the cyclase enzymatic domain. Additionally, two specific c-di-AMP PDEs have been discovered in bacteria: one containing a DHH-DHHA1 domain (Romling, 2008; Corrigan and Grundling, 2013), a class which is likely not present in cyanobacteria; the other with a domain architecture similar to the 7TM_7TMR_HD protein family (Huynh et al., 2015). The 7TM_7TMR_HD is more common than DHH-DHHA1 domain-containing PDEs in bacteria and it is also present in cyanobacterial genomes (Huynh et al., 2015; Huynh and Woodward, 2016).

Recently, an assessment of regulons of riboswitches involved in binding the second messenger c-di-AMP suggested a function of c-di-AMP in regulating the synthesis of osmoprotectants under abiotic stress in cyanobacteria (Nelson et al., 2013). The addition of organic solutes, which are not permeable to the bacterial cell, to cellular growth medium induces osmotic stress. Although salt stress is often referred to as osmotic stress (Hagemann, 2011), salt stress specifically results in reduced water availability due to dissolved ions that concomitantly induce osmotic stress (Pade and Hagemann, 2015). Thus, salt stress includes both ionic stress and secondary osmotic stress. The primary signals induced in response to salt and osmotic stress in cyanobacteria are still being elucidated (Pade and Hagemann, 2015). However, a role for two component histidine kinase Hik33 in responses to osmotic and salt stress in cyanobacteria has been noted (Mikami et al., 2002; Marin et al., 2003; Paithoonrangsarid et al., 2004; Shoumskaya et al., 2005). The primary abiotic stress can also induce secondary signals, including second messengers. In cyanobacteria, the second messenger Ca^2+^ is involved in organismal responses to environmental osmotic and salt changes (Torrecilla et al., 2001). The recognition that potential c-di-AMP binding riboswitches may be involved in osmoprotectant production in response to osmotic stress in cyanobacteria suggests a potential role for c-di-AMP in cellular responses to abiotic stress.

In this study, we investigated the activity of DAC and PDE enzymes in the moderately halotolerant freshwater unicellular cyanobacterium *Synechocystis*, the potential for abiotic stresses to alter intracellular c-di-AMP levels, and the impact of altering c-di-AMP homeostasis on the physiology and survival of this organism. Furthermore, we assessed the impacts of osmotic and salt stresses on modulating c-di-AMP homeostasis in several additional cyanobacteria, including another freshwater unicellular strain *Synechococcus elongatus* PCC 7942 (hereafter *Synechococcus*) for which c-di-AMP has been recently implicated in nighttime survival (Rubin et al., 2018), and two filamentous freshwater strains *Fremyella diplosiphon* [also known as *Tolypothrix* sp. PCC 7601 (Yerrapragada et al., 2015)], and *Anabaena* sp. PCC 7120 (hereafter *Anabaena*, also known as *Nostoc* sp. PCC 7120) to more broadly understand the modulation of c-di-AMP homeostasis across a range of cyanobacteria.

## Materials and Methods

### Plasmid Construction in *Synechocystis*

Intracellular levels of c-di-AMP in *Synechocystis* were targeted for increase by overexpressing the native DAC protein Sll0505 or reduction by overexpressing the native PDE protein Slr0104. The open reading frame of native genes encoding the DAC and PDE enzymes were constitutively overexpressed under the control of the *apcE* (*slr0335*) promoter using the self-replicating plasmid pRL1342 (Wolk et al., 2007; GenBank: AF403427.1). Promoters were added to the DAC- or PDE-encoding genes by overlap PCR using primers indicated in **Table 1**. The genes were amplified from genomic DNA with PrimeSTAR Max DNA polymerase (Clontech Laboratory, Inc.) using primers that encoded *Xho*I and *BamH*I restriction sites (**Table 1**). The promoter-gene fusion product and pRL1342 were restricted with *Xho*I and *BamH*I and the cleaved products ligated using DNA Ligation Kit, Mighty Mix (Takara). After transformation of the ligation mix into *E. coli* DH5α competent cells (Life Technologies, Inc.), transformants were selected on LB agar containing chloramphenicol at 50 μg mL^-1^ (w/v). The DNA sequences of isolated plasmids were confirmed by Sanger sequencing. The plasmid was then inserted into *Synechocystis* by triparental mating as previously described (Agostoni et al., 2016).

**Table 1:** Primers used in this study.

	Forward primer (5′-3′)^a^	Reverse primer (5′-3′)	Purpose
OE*sll0505*_np*apcE*	CGCG**CTCGAG**TTAAAACTGCATTATCAG	CTGTCAATGGCGACTCCCCGATTGAGGAAA	DAC cloning
OE*sll0505*	TTTCCTCAATCGGGGAGTCGCCATTGACAG	CGC**GGATCC**TCATTTTTTGTCGTT	DAC cloning
OE*slr0104*_np*apcE*	CGCG**CTCGAG**TTAAAACTGCATTATCAG	GGCAAAAATTGCTTTCATTGGATTTCATTATCTCCC	PDE cloning
OE*slr0104*	GGGAGATAATGAAATCCAATGAAAGCAATTTTTGCC	CTC**GGATCC**CTAAAATCTGGTGGTG	PDE cloning
*sll0504*	ACCGGATGAACGACGAAATTA	TAGACAATCCTGGCGCAATAG	RT-PCR/qRT-PCR
*sll0505*	GGAGTCGCCATTGACAGTAA	TCCTCGGAAACGACAATACAA	RT-PCR/qRT-PCR
*sll0506*	CCGGATTTGGACCAGCA	TCCTTTAATTCCCGCCGTAG	RT-PCR/qRT-PCR
*slr0104*	CGCCCAACTCAAACAAGAAAG	GTTGCTGCTCCAGGGTAAA	RT-PCR/qRT-PCR
*rnpB*	GTGAGGACAGTGCCACAGAA	GGCAGGAAAAAGACCAACCT	qRT-PCR

^a^Bold text indicates sequence of restriction site.

### Culture Conditions

Axenic cultures of *Synechocystis*, *F. diplosiphon*, *Synechococcus*, and *Anabaena* were grown at 28°C in BG-11 (Allen, 1968) containing 20 mM HEPES at pH 8.0 (hereafter BG-11/HEPES) with the indicated antibiotic when needed. *F. diplosiphon* strain SF33, a shortened-filament mutant strain that displays wild-type (WT) pigmentation (Cobley et al., 1993), was used as the WT *F. diplosiphon* strain. Cultures (25 ml) in 250 ml glass flasks were adapted to fluorescent white light (WL; Philips F32T8/TL741/ALTO) at 15 μmol m^-2^ s^-1^ with shaking at 175 rpm for at least a week. Growth rate of the WT, overexpression (OE) DAC, and OE PDE strains was estimated by optical density at 750 nm (OD_750_) every day for strains grown under WL (Philips F32T8/TL741/ALTO) at 35 μmol m^-2^ s^-1^ with shaking at 175 rpm. A secondary analysis of growth was conducted on BG-11 plates containing 1% (w/v) agar. A 1:10 dilution series of cells growing homogenously in liquid culture was plated for each strain with the initial cultures at an OD_750_ of 0.6. Aliquots of 10 μL of the dilutions up to 1:10,000 were plated and the plates incubated under 15 μmol m^-2^ s^-1^ of WL.

### Abiotic Stresses and c-di-AMP/c-di-GMP Quantification

Cells were grown to an optical density at 750 nm (OD_750_) of 1 and transferred to new 250 ml flasks with BG-11/HEPES medium containing 0.2 M sorbitol for the osmotic stress, or 0.2 M NaCl for the ionic stress, except for halophile *Synechocystis* for which the NaCl concentration was 0.6 M. As a control, BG-11/HEPES medium without sorbitol or NaCl added was utilized. Cells were maintained under osmotic or salt stress for 24 h. After 24 h, c-di-AMP and c-di-GMP were quantified as described (Massie et al., 2012; Agostoni et al., 2013; Barker et al., 2013). In brief, c-di-AMP and c-di-GMP were quantified by UPLC-MS/MS. Prior to analysis, an aliquot of each sample was dried under vacuum to remove extraction buffer and the pellet was resuspended in an equal volume of water. A 10-μl volume of the resuspended sample was analyzed together with an eight-point standard curve of purified c-di-AMP or c-di-GMP (Biolog). C-di-AMP and c-di-GMP concentrations determined for samples were normalized to total soluble protein content from an equal volume of cells from which second messengers were extracted as previously described (Agostoni et al., 2013; Zhu et al., 2016). Growth over time of WT, OE DAC and OE PDE *Synechocystis* stains in the presence of sorbitol (0.5 M) or NaCl (0.6 M) was measured using OD_750_ as described above.

### Quantitative Reverse Transcriptase PCR (qRT-PCR) and RT-PCR in *Synechocystis*

For RNA extraction, *Synechocystis* cells from a 10 ml aliquot of culture were collected 24 h after the osmotic stress or after subculturing. RNA was isolated using Trizol reagent essentially as described (Seib and Kehoe, 2002; Singh and Montgomery, 2013a). Total RNA extracted was treated with a TURBO DNA-free kit (Ambion, Austin, TX, United States). cDNA synthesis was performed as described (Pattanaik and Montgomery, 2010) with 0.5 μg of total RNA using the Reverse Transcription System (Promega Corporation, Madison, WI, United States). Control reactions were conducted in which no reverse transcriptase (No RT) was added to the reaction mixtures. Gene *rnpB* (RNase P subunit B), the expression of which is not altered by nutrient or salt stress (Kloft et al., 2005; Zhang et al., 2007; Wang et al., 2014), was used as an internal control. RT cycling parameters were denaturing at 95°C for 20 s, 40 cycles of denaturation at 95°C for 3 s and annealing/extension at 60°C for 30 s, followed by melt-curve analysis starting at 60°C and ending at 95°C for 15 min. **Table 1** shows primers used for qRT-PCR and RT-PCR.

### Genome Comparisons

Phylogenetic analyses of multiple conserved DAC domain sequences (PF02457) from 83 finished cyanobacterial genomes present in the IMG database were performed using SeaView4 software (Galtier et al., 1996). Multiple alignments of amino acid sequences were generated using MUSCLE (Edgar, 2004). Phylogenetic trees were inferred using maximum likelihood-based method 100 bootstraps, and the Jones-Taylor-Thornton model (Jones et al., 1992). The likelihood log was -23144.7.

### Cell Lysis Assay

The cell lysis assay was conducted by pelleting 7 mL of cells that had been grown in BG-11/HEPES medium under WL at 15 μmol m^-2^ s^-1^ as described above and diluted to an OD_750_ of 0.4 and incubated for an additional 24 h. Pelleted cells were then resuspended in 4 mL of CelLytic^TM^ B (Millipore Sigma, St. Louis, MO, United States) containing 10 μg/mL (w/v) lysozyme and shaken on a vortexer for 30 min. After this period, samples were centrifuged at 10,000 ×*g* at room temperature for 5 min. The level of cellular lysis was estimated based on measuring absorbance at 660 nm of the chlorophyll released into the supernatant (Mehta et al., 2015). Chlorophyll absorbance values were standardized relative to total soluble protein content of cells.

### Statistical Analysis

Experiments were conducted with at least three independent biological replicates. Statistical significance was determined via Student’s *t*-test or via one way analysis of variance (ANOVA) with Fisher *post hoc* test using OpenStat statistical software (version 10.01.08; W. G. Miller http://www.Statprograms4U.com). Statistical analyses were performed utilizing 95% confidence intervals (*p* < 0.05).

## Results

### Bioinformatic and Evolutionary Analyses of c-di-AMP Synthesis Genes in Cyanobacteria

**FIGURE 1:**
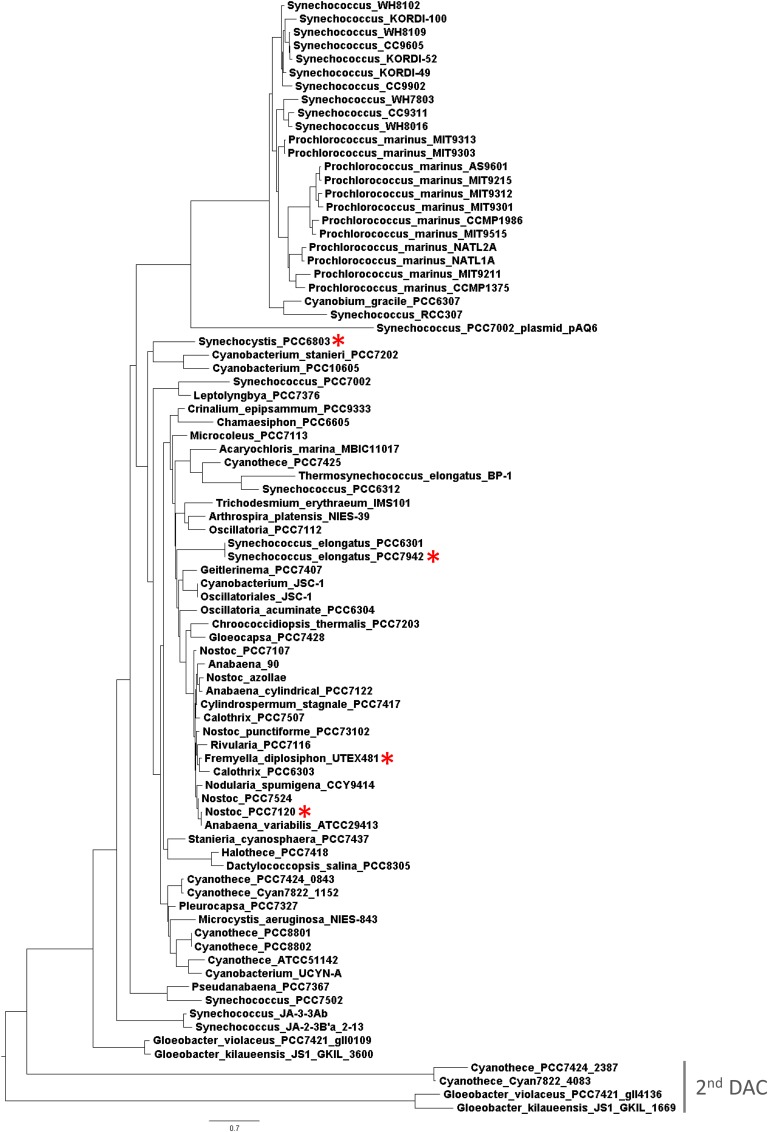
Phylogenetic analysis based on multiple putative functionally conserved DAC sequences. For the species with two DACs (see gray line for the second rare DAC), the gene identification number was added. Red asterisks indicate four species investigated in this study.

Nearly all sequenced cyanobacteria assessed contain one copy of the c-di-AMP synthesis gene DAC, with the exception of *Cyanothece* sp. PCC 7424, *Cyanothece* sp. PCC 7822, *Gloeobacter kilaueensis* JS1, and *Gloeobacter violaceus* PCC 7421, which each carry two copies (Agostoni and Montgomery, 2014). Notably, DACs from cyanobacteria lack additional sensor domains (Agostoni and Montgomery, 2014), in contrast to DACs from other bacteria that contain domains involved in multimerization or in regulating enzymatic activity (Corrigan and Grundling, 2013; Commichau et al., 2015). The genus *Gloeobacter*, which represents primordial cyanobacteria (Turner et al., 1999), possesses both DACs suggesting that c-di-AMP signaling was present early during the evolution of this phylum. Phylogenetic analysis based on amino acid sequences of DACs in cyanobacteria (**Figure 1**) indicated that DACs have been vertically transferred as the typology of the tree is similar to one generated based on phylogenetic diversity of cyanobacterial genomes (Shih et al., 2013). For cyanobacterial species with two DACs, one of the two copies is extremely divergent from that of all the other DACs found in species with just one copy (**Figure 1**, indicated as “2^nd^ DAC”). The diverged copy of DAC is related to other unknown or hypothetical DAC enzyme-encoding genes that contain a DisA-N domain, which may indicate a novel class of DAC enzymes.

**FIGURE 2:**
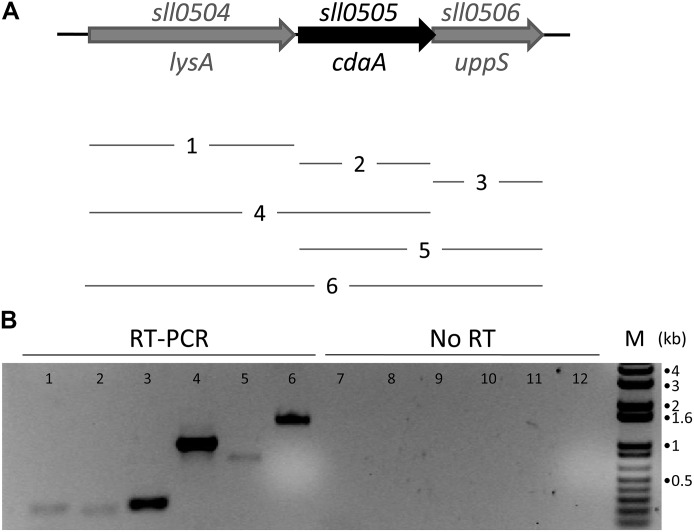
Genomic context and RT-PCR analyses of conserved DAC-containing operon in *Synechocystis*. **(A)** Genomic context of conserved DAC (*sll0505*) in *Synechocystis*. Lines (1–6) below genomic region indicate PCR products amplified from cDNA in panel B. RT-PCR of DAPDC (*sll0504*), DAC (*sll0505*), and UPS (*sll0506*) in *Synechocystis*. **(B)** RT-PCR analyses of transcripts produced from the DAC genomic region in *Synechocystis*: Lanes 1 and 7, DAPDC; Lanes 2 and 8, DAC; Lanes 3 and 9, UPS; Lanes 4 and 10, DAPDC and DAC; Lanes 5 and 11, DAC and UPS; Lanes 6 and 12, DAPDC, DAC, and UPS. Lanes 1–6, RT-PCR reactions; Lanes 7–12, no RT negative control reactions. M, molecular marker. RNA was isolated from cells grown at 35 μmol m^-2^ s^-1^ white light.

Cyanobacteria exhibit a conserved operon structure for the DAC gene related to *cdaA*/*dacA* that is found broadly across distinct strains. The Diaminopimelate Decarboxylase (DAPDC or *lysA*) and Undecaprenyl Pyrophosphate Synthase (UPS or *uppS*) genes are always downstream and upstream, respectively, from the DAC gene which is conserved across cyanobacteria (**Figure 2A**). It appears that the grouping of these three genes in an operon is a unique feature of cyanobacteria, as this arrangement is absent in non-cyanobacterial species. In species with two DACs, only one DAC is found in this gene arrangement (data not shown). DAPDC catalyzes the last step in the biosynthesis of the amino acid lysine, i.e., conversion of the peptidoglycan precursor DAP to lysine (Bukhari and Taylor, 1971a,b). UPS is a prenyltransferase that catalyzes the production of undecaprenyl pyrophosphate, which is critical as a lipid carrier for peptidoglycan synthesis (Apfel et al., 1999). Thus, both neighboring genes appear to be implicated in peptidoglycan synthesis. In *Synechocystis*, there is a 42 bp intergenic region between the DAPDC gene (*sll0504*) and DAC gene (*sll0505*), whereas the DAC overlaps with the UPS gene (*sll0506*). We decided to verify whether the three genes were co-transcribed. RT-PCR analyses of amplicons of portions of the mRNA indicated that the three genes can be transcribed together in a single operon in *Synechocystis* (**Figure 2B**, lane 6), suggesting that they may be involved in similar or related functions in the organism.

### Overexpression of Native DAC-Encoding *sll0505* and PDE-Encoding *slr0104* Genes in *Synechocystis*

**FIGURE 3:**
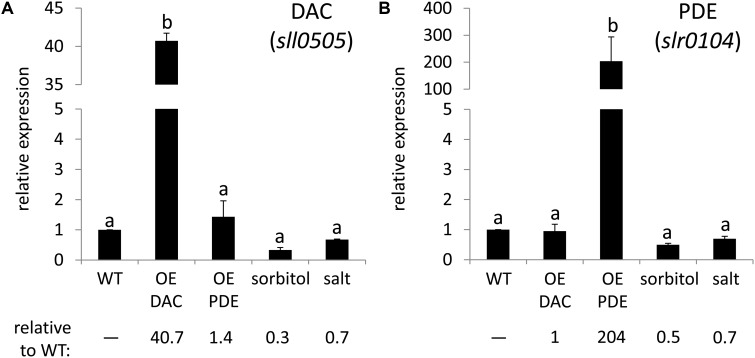
Quantitative reverse transcriptase PCR (qRT-PCR) analysis of the expression of DAC and PDE genes in *Synechocystis*. **(A)** DAC and **(B)** PDE genes were analyzed for strains grown under 35 μmol m^-2^ s^-1^ white light. Wild-type (WT), overexpression of DAC (*sll0505*) in WT (OE DAC), overexpression of PDE (*slr0104*) in WT (OE PDE), WT under sorbitol stress (sorbitol, 0.2 M sorbitol), WT under salt stress (salt, 0.6 M NaCl). The transcript level of the *rnpB* gene was used as an internal control for each sample. Bars represent averages (± standard deviations). Bars marked with different letters are significantly different (*p* < 0.05). Numbers below graphs represent fold difference relative to WT grown under non-stress conditions.

In *Synechocystis* there is only one DAC (PF02457, *sll0505*), which is related to *cdaA*/*dacA*, and one c-di-AMP PDE (PF07698, *slr0104*, belonging to the 7TM_7TMR_HD family), which is related to *pgpH*. In many bacteria c-di-AMP is essential for survival (Commichau et al., 2015), and in accordance with this observation, we were not able to produce a mutant completely lacking *sll0505* in *Synechocystis* (data not shown). We, thus, decided to overexpress the c-di-AMP DAC and PDE native enzymes in *Synechocystis* to investigate their activity *in vivo*. Quantitative RT-PCR showed increased accumulation of the mRNA for DAC and PDE genes in the DAC and PDE overexpression strains, respectively (**Figure 3**). The DAC strain exhibited an ∼40-fold increase in the level of DAC mRNA accumulation compared to WT (**Figure 3A**), whereas the PDE strain had a more than 200-fold increase in PDE mRNA levels compared to WT (**Figure 3B**).

**FIGURE 4:**
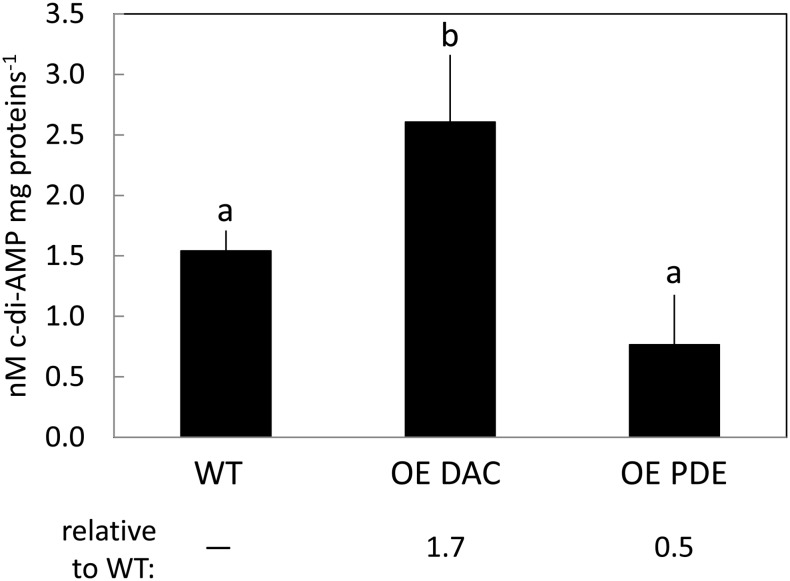
Cyclic di-AMP levels in *Synechocystis* in replete BG-11/HEPES medium under white light. WT, wild-type; OE DAC, strain overexpressing the native DAC (*sll0505*); OE PDE, strain overexpressing the native PDE (*slr0104*). Bars represent averages (± standard deviations). Bars marked with different letters are significantly different (*p* < 0.05). Numbers below graphs represent fold difference relative to WT.

Quantification of c-di-AMP levels in the DAC and PDE overexpression strains was conducted to assess whether these two native enzymes could modulate intracellular levels of c-di-AMP (**Figure 4**). The levels of c-di-AMP in the DAC overexpression strain were significantly altered, i.e., 1.7-fold higher (*p* < 0.05), compared to the WT strain. Whereas this is lower than might be anticipated based on gene expression data, some bacterial strains which overaccumulate DAC mRNA show no upregulation of intracellular c-di-AMP levels suggesting possible negative feedback mechanisms on DAC activity in some cases (Savage et al., 2015). The levels of c-di-AMP in the PDE overexpression *Synechocystis* strain were on average half as much as the WT strain, although this level was not significantly different. To assess whether the overexpression of the DAC and c-di-AMP-specific PDE were specific to affecting c-di-AMP levels, we assessed levels of another second messenger c-di-GMP. We observed no difference in c-di-GMP levels between WT, DAC-overexpression strain, and c-di-AMP PDE overexpression strain (data not shown).

**FIGURE 5:**
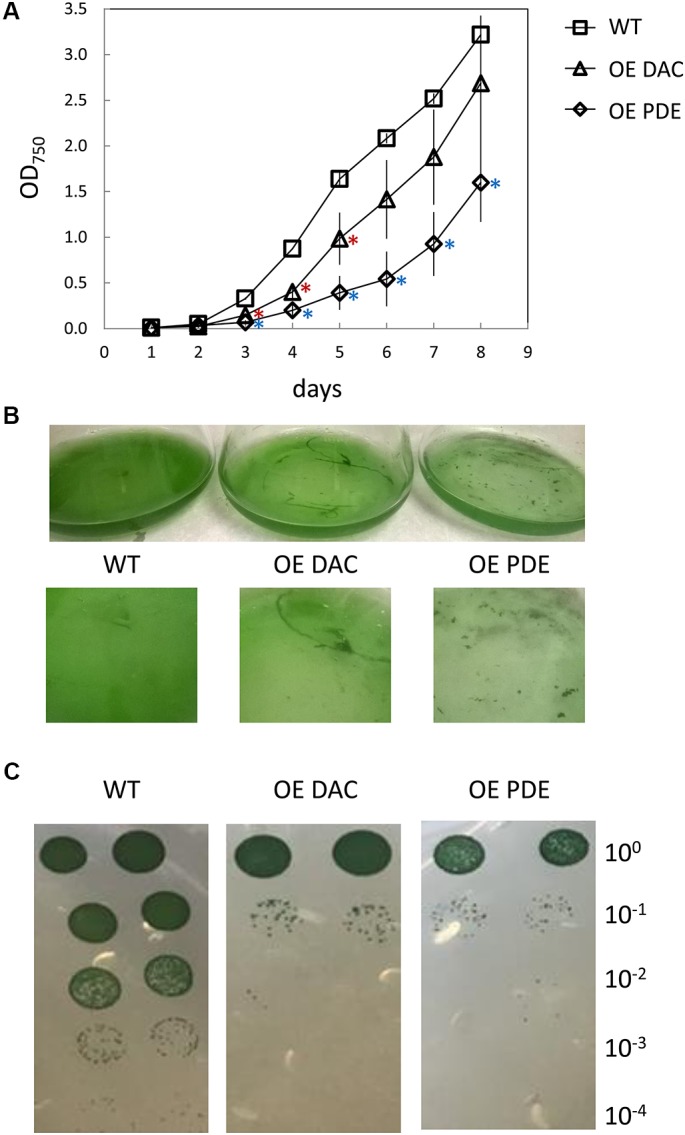
Growth curve of *Synechocystis* wild-type (WT), strain overexpressing diadenylyl cyclase (OE DAC), and strain overexpressing c-di-AMP phosphodiesterase (OE PDE). **(A)** All strains were grown in BG-11/HEPES under 35 μmol m^-2^ s^-1^ white light. Data points represent averages (± standard deviations). ^∗^*p* < 0.05 with red asterisks indicating comparison between OE DAC and WT and blue asterisks comparing OE PDE and WT. **(B)** Picture of representative WT, OE DAC, and OE PDE cultures. **(C)** Images of WT, OE DAC, and OE PDE growth as measured by spotting of cells (10 μL) of a 1:10 serial dilution on BG-11/HEPES plates containing 1% (w/v) agar under white light. Cells were initially at an OD_750_ of 0.6 in the undiluted culture and cells diluted up to 1:10,000-fold.

Previously, it has been observed that c-di-AMP homeostasis is fundamental for optimal growth, with either lower than normal levels or overaccumulation relative to WT resulting in defects in growth (Corrigan et al., 2013; Mehne et al., 2013; Ye et al., 2014; Gundlach et al., 2015; Rubin et al., 2018). Our overexpressing strains both exhibited a lag in growth compared to WT in BG-11/HEPES medium (**Figure 5A**). Additionally, WT grew homogenously in the medium, whereas DAC and PDE strains formed distinct aggregates in the late part of the growth curve analysis (**Figure 5B**). This aggregation may impact analysis of growth by measuring culture optical density. Thus, we also assessed growth using dilution-based colony growth assays on agar plates. In this assay, we similarly observed that the OE DAC and OE PDE strains exhibited impaired growth relative to WT (**Figure 5C**).

**FIGURE 6:**
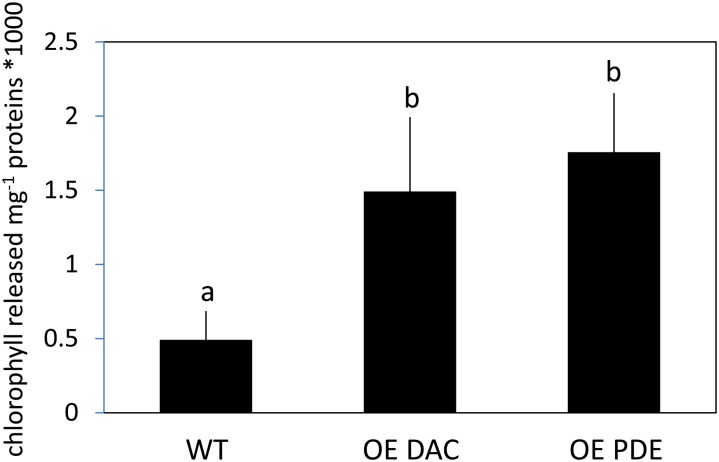
Comparison of lysozyme-dependent lysis of *Synechocystis* wild-type (WT), strain overexpressing diadenylyl cyclase (OE DAC), and strain overexpressing c-di-AMP phosphodiesterase (OE PDE). All strains were grown in BG-11/HEPES under 15 μmol m^-2^ s^-1^ white light. Cells were treated with 10 μg mL^-1^ lysozyme for 30 min. Cellular lysis was measured based on chlorophyll released from cells at measured by absorbance at 660 nm in the supernatant after centrifugation. Bars represent averages (± standard deviations). Bars marked with different letters are significantly different (*p* < 0.05).

Given the association of the c-di-AMP synthesis gene with other genes associated with peptidoglycan synthesis or modification and the cellular aggregation phenotypes, c-di-AMP accumulation in cells may impact cell wall synthesis or modification. We, thus, tested for alterations in cell wall properties for the OE strains relative to WT by conducting lysozyme sensitivity assays. Both the OE DAC strain and OE PDE strain had increased sensitivity to lysozyme treatment relative to WT, as measured by chlorophyll release into the supernatant (**Figure 6**).

### Osmotic and Salt Stresses Impact c-di-AMP Levels in Multiple Cyanobacteria

Based on the observation that c-di-AMP-binding riboswitches have a putative role in osmoprotectant synthesis and transport in cyanobacteria (Nelson et al., 2013), c-di-AMP could be critical for osmotic or salt stress responses in these organisms. Osmoprotectants have recognized roles in cellular responses to both osmotic and salt stresses (Bougouffa et al., 2014). Thus, we quantified changes in DAC and PDE mRNA levels in WT under osmotic and salt stresses, the latter of which induces both osmotic and ionic stress. After 24 h of osmotic or salt stress, levels of DAC and PDE mRNA decreased compared to non-stress, control conditions although differences were not statistically significant (**Figure 3**). The DAC mRNA levels decreased to 0.3- and 0.7-fold relative to WT under sorbitol and salt stress, respectively (**Figure 3A**). Similarly, the PDE mRNA levels decreased to 0.5- and 0.7-fold relative to WT under sorbitol and salt stress, respectively (**Figure 3B**).

**FIGURE 7:**
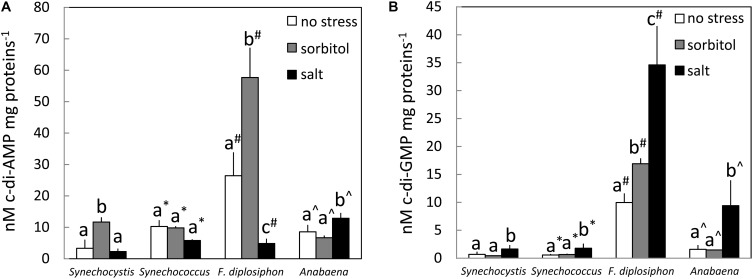
Cyclic di-AMP and cyclic di-GMP levels under sorbitol and salt stresses. **(A)** Cyclic di-AMP and **(B)** cyclic di-GMP levels normalized to total proteins were determined for *Synechocystis* sp. PCC 6803 (*Synechocystis*), *Synechococcus elongatus* sp. PCC 7942 (*Synechococcus*), *Fremyella* *diplosiphon* (*F. diplosiphon*), and *Anabaena* sp. PCC 7120 (*Anabaena*) grown under 35 μmol m^-2^ s^-1^ white light. Bars represent averages (± standard deviations). Bars marked with different letters indicate a significant difference (*p* < 0.05) for comparisons made within each species.

To determine whether these stresses influence intracellular c-di-AMP levels and whether stress-induced changes occur in cyanobacteria beyond *Synechocystis*, we exposed *Synechocystis* and three additional species of cyanobacteria to sorbitol or salt stress for 24 h. Two were unicellular cyanobacteria, moderately halotolerant *Synechocystis* (Reed et al., 1985) and salt-sensitive *Synechococcus* (Kaku et al., 2000), and two were filamentous cyanobacteria, highly salt-sensitive *Anabaena* (Rai and Tiwari, 2001) and salt-sensitive *F. diplosiphon* (Singh and Montgomery, 2013b). C-di-AMP levels were threefold higher under sorbitol-induced osmotic stress in *Synechocystis*. By comparison, there was no significant difference in intracellular levels of c-di-AMP in another unicellular strain *Synechococcus* in response to osmotic stress (**Figure 7A**). In the filamentous cyanobacterium *F. diplosiphon*, c-di-AMP levels were much higher than for the other cyanobacterial strains and increased ∼2-fold under osmotic stress, whereas sorbitol treatment did not result in an increase in c-di-AMP under our conditions in *Anabaena* (**Figure 7A**). Changes in the intracellular concentration of c-di-AMP occur during osmotic stress in *Synechocystis* and *F. diplosiphon*. We also investigated whether c-di-AMP levels varied in these four cyanobacterial species under salt stress (**Figure 7A**). Under salt stress, c-di-AMP levels were not impacted in unicellular strains, whereas levels were lower in *F. diplosiphon* and higher in *Anabaena* in the presence of salt.

In cyanobacteria, biofilm formation is a protective mechanism against salt stress (Jittawuttipoka et al., 2013). We previously demonstrated that induction of biofilm formation is under the control of c-di-GMP in *Synechocystis* (Agostoni et al., 2016). To investigate whether c-di-GMP is elevated under salt stress conditions that are associated with cyanobacterial biofilm formation, we quantified c-di-GMP levels in response to treatment with salt. Indeed, c-di-GMP levels increased in all four species after 24 h of salt stress (**Figure 7B**). In contrast to stress caused by salt, the levels of c-di-GMP did not vary in *Synechocystis*, *Synechococcus*, and *Anabaena* under sorbitol stress (**Figure 7B**). However, c-di-GMP levels significantly increased under sorbitol stress in *F. diplosiphon* (**Figure 7B**). Taken together, these analyses suggest that c-di-AMP responds to osmotic and salt stress, although generally an increase in c-di-AMP levels is associated with sorbitol-induced osmotic stress (if there is a response). By contrast, levels of c-di-GMP primarily respond to ionic rather than osmotic stress across cyanobacterial species, with an increase in c-di-GMP levels observed in all tested strains in response to salt stress. The distinct responses of these cyanobacterial strains to salt and osmotic stresses may reflect their distinct sensitivities to salt (Reed et al., 1985; Kaku et al., 2000; Rai and Tiwari, 2001; Singh and Montgomery, 2013b), as well as other aspects of their unique ecological histories. However, the production of c-di-AMP in a range of cyanobacterial species is evident.

**FIGURE 8:**
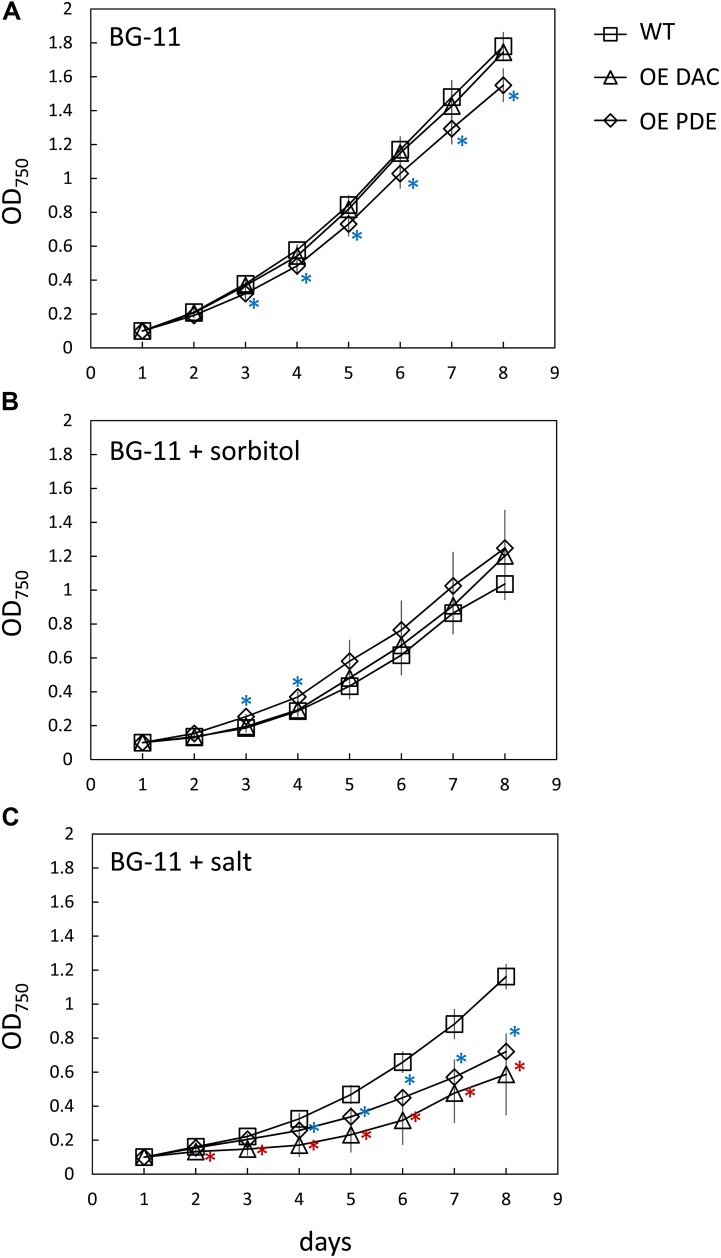
Growth curve of *Synechocystis* wild-type (WT), strain overexpressing diadenylyl cyclase (OE DAC), and strain overexpressing c-di-AMP phosphodiesterase (OE PDE) under low white light and in presence of abiotic stresses. All strains were grown in BG-11/HEPES under 15 μmol m^-2^ s^-1^ white light in **(A)** BG-11 medium, **(B)** sorbitol-induced osmotic stress (0.5 M sorbitol), or **(C)** salt stress (0.6 M NaCl). Data points represent averages (± standard deviations). ^∗^*p* < 0.05 with red asterisks indicating comparison between OE DAC and WT and blue asterisks comparing OE PDE and WT.

**FIGURE 9:**
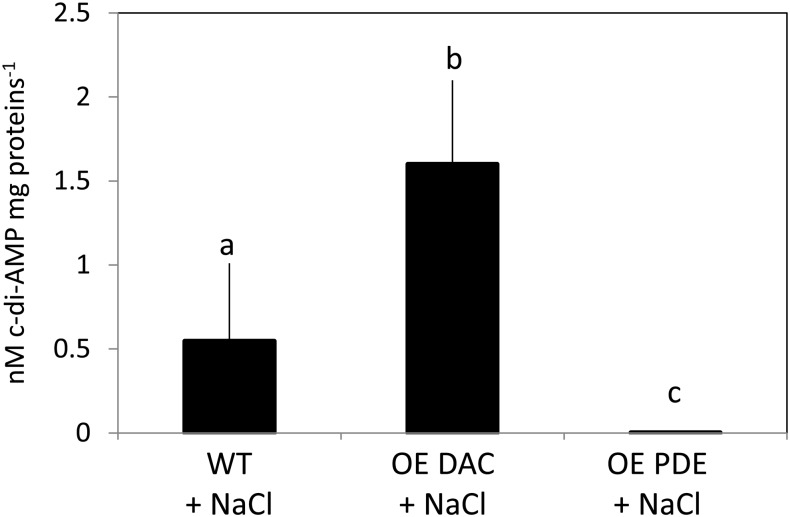
Cyclic di-AMP levels in *Synechocystis* in replete BG-11/HEPES medium containing 0.6 M NaCl under low white light at 15 μmol m^-2^ s^-1^. WT, wild-type; OE DAC, strain overexpressing the native DAC (*sll0505*); OE PDE, strain overexpressing the native PDE (*slr0104*). Bars represent averages (± standard deviations). Bars marked with different letters are significantly different (*p* < 0.05).

Given that at least osmotic stress results in an alteration of intracellular c-di-AMP levels in *Synechocystis*, we queried whether strains with overexpression of DAC or PDE genes exhibited altered growth responses due to applied abiotic stresses. In these analyses, we reduced the intensity of white light to which cells were exposed (compared to results shown in **Figure 5**) due to the noted combined detrimental effect of multiple stresses such as high light and salt on cyanobacterial growth (Lu and Zhang, 2000). We first noted that, although not directly comparable due to changing multiple factors, growth under lower light (i.e., ∼15 μmol m^-2^ s^-1^) resulted in a significant impairment only of the OE PDE strain compared to WT compared to growth under higher white light (i.e., 35 μmol m^-2^ s^-1^). Additionally, OE DAC and OE PDE cells exposed to low light lacked a major lag in growth relative to WT that was observed at higher white light levels (compare **Figure 5A** and **Figure 8A**). Osmotic stress conditions had minor effects, with only the OE PDE strain having statistically significant, transiently improved growth relative to WT (**Figure 8B**). However, both OE PDE and OE DAC strains exhibited an impairment in growth relative to WT in the presence of salt (**Figure 8C**). The significant changes in growth under salt in the OE DAC and OE PDE strains relative to WT were associated with altered intracellular c-di-AMP levels. The OE DAC strain exhibited significantly higher intracellular c-di-AMP levels compared to WT, whereas c-di-AMP levels in the OE PDE strain were below detection when strains were grown in the presence of NaCl (**Figure 9**).

## Discussion

Given that the genus *Gloeobacter*, which is considered the most primordial of extant cyanobacteria (Rippka et al., 1974), possesses two DAC enzymes, in contrast with the majority of cyanobacterial species that have only have one DAC enzyme, we speculate that cyanobacteria initially contained two DAC enzymes and that during evolution the second DAC was lost. Since the conserved DAC, DAPDC and the UPS cassette (i.e., *lysA*-*cdaA*-*uppS* operon) is extremely conserved among cyanobacteria, uniquely, and both the DAC and UPS have associations with peptidoglycan synthesis, these three genes together likely play a critical role in controlling cell wall synthesis across cyanobacteria. This is a role consistent with prior studies which indicated that c-di-AMP metabolism impacts cell wall structure or stability in multiple, largely pathogenic, bacteria (Commichau et al., 2017). However, the conserved operon structure suggest an important and well-conserved function for c-di-AMP in peptidoglycan-dependent processes in cyanobacteria.

We were able to strongly increase transcript accumulation of DAC and PDE in *Synechocystis* using overexpression plasmids and demonstrate a significant impact on intracellular c-di-AMP levels in the OE DAC strain in BG-11 and significant impacts on c-di-AMP homeostasis in both the OE DAC and OE PDE strains grown in the presence of salt. Higher or lower levels of c-di-AMP are both detrimental to normal growth in *Bacillus subtilis*, *Listeria monocytogenes, Borrelia burgdorferi*, and *Staphylococcus aureus* (Corrigan et al., 2011; Dengler et al., 2013; Mehne et al., 2013; Witte et al., 2013; Ye et al., 2014). Also, reduced levels of c-di-AMP have been recently shown to impact cyanobacterial growth in a *Synechococcus* strain in which *cdaA* was deleted (Rubin et al., 2018). In *B. subtilis* the differences in growth rates between WT and a strain with strong accumulation of c-di-AMP were attributed to aberrant cell morphologies (Mehne et al., 2013). Given that the *cdaA* gene is next to a gene encoding GlmM that is essential for peptidoglycan synthesis in *B. subtilis*, the observed aberrant cell morphologies may be due to disruptions in c-di-AMP regulation of peptidogylan synthesis in the mutant compared to WT (Mehne et al., 2013). Mutation of the *S. aureus* PDE GdpP resulted in increased peptidoglycan cross-linking which was detrimental to growth (Corrigan et al., 2011).

Here, *Synechocystis* strains with overexpression of either DAC or PDE grew slower than WT. Notably, the DAC and PDE overexpression strains formed aggregates later in the growth analysis compared to WT which grew homogenously in the medium throughout. These phenotypes may be related to the observed changes in cell morphology or cell wall integrity of c-di-AMP mutants in several bacteria (Corrigan et al., 2011; Bai et al., 2013; Mehne et al., 2013; Witte et al., 2013; Zhang and He, 2013; Tang et al., 2015). Indeed, the DAC and PDE overexpression strains exhibited increased sensitivity to lysozyme as compared to the WT parent strain. The results for the OE PDE strain are in accordance with prior analyses showing that decreased c-di-AMP levels have previously been implicated in cell wall sensitivity and altered cell lysis for a number of bacteria (Luo and Helmann, 2011; Witte et al., 2013). On the other hand, the increased sensitivity observed for the OE DAC strain may be associated with the fact that the DAC/*cdaA* gene is in an operon with genes associated with cell wall synthesis/modification. Specifically, the role of the DADPC gene in crosslinking of peptidoglycan corresponds to prior associations of c-di-AMP levels with peptidoglycan cross-linking (Luo and Helmann, 2011). Thus, the altered phenotype of the OE DAC strain could be related to associated impacts of altering c-di-AMP accumulation on peptidoglycan crosslinking, which in turn could alter cellular responses to lysozyme.

Cyclic di-AMP homeostasis is critical in replete BG-11 medium. Additionally, the regulation of DAC and PDE genes in osmotic and salt stress, especially the latter, appears to contribute to cell fitness. Whereas WT has impaired growth in both stresses, changes in intracellular c-di-AMP homeostasis is important for cellular responses to salt stress as both the DAC and PDE OE strains, which have significantly higher and lower c-di-AMP levels in the presence of salt, respectively, exhibited impaired growth over time in the presence of NaCl-induced stress.

Osmotic and ionic stresses are common in natural ecosystems (Williams, 1987; Kaushal et al., 2005; Durack et al., 2012; Canedo-Arguelles et al., 2013; El-Akhal et al., 2013) and identifying the mechanisms by which cyanobacteria can tolerate osmotic and ionic stresses is critical. Species able to maintain osmotic equilibrium under these conditions will prove most beneficial for use in cyanobacterial mass cultivation (Hagemann, 2011). There is still a lack of knowledge on the mechanisms used by cyanobacteria to specifically sense and respond to osmotic and ionic stresses. Experiments described here show that sorbitol is an important factor in the regulation of c-di-AMP homeostasis, whereas salt is a critical factor to regulate c-di-AMP synthesis and c-di-GMP homeostasis. Prior studies with Gram-positive bacteria have implicated c-di-AMP as critical during osmotic stress, based on the identification of a potassium transporter and a regulator of a K^+^ transporter as c-di-AMP receptors (Corrigan et al., 2011, 2013; Bai et al., 2014; Moscoso et al., 2015). Also, regulation of c-di-AMP levels have been associated with salt sensitivity in Gram-positive strains (Smith et al., 2012; Dengler et al., 2013). Notably, a study on c-di-AMP-binding riboswitches implicated c-di-AMP-dependent regulation of the synthesis and transport of osmoprotectants in cyanobacteria as critical for stress responses, such as osmotic or salt stresses, in these organisms (Nelson et al., 2013). Our results suggest a role in response to salt stress in *Synechocystis*. Determining the molecular mechanisms of c-di-AMP and c-di-GMP signaling networks during cyanobacterial adaptation is necessary to understand how cyanobacteria survive in stressful and fluctuating environments and ensure improved biomass and product yields under osmotic and ionic stresses to improve applications and fundamental research in solving environmental problems.

## Author Contributions

MA and BM: conceived and designed the experiments. MA, AL-J, EH, GS, and EB: performed the experiments and contributed to data analysis. MA and BM: drafted the article. MA, AL-J, CW, and BM: critically revised the article.

## Conflict of Interest Statement

The authors declare that the research was conducted in the absence of any commercial or financial relationships that could be construed as a potential conflict of interest. The reviewer RS and handling Editor declared their shared affiliation.
